# Evaluation of the Profile of Selected Bioactive Compounds and the Potential of Barley Wort Enriched with Malted and Unmalted Hemp Seeds for Brewing Applications

**DOI:** 10.3390/molecules30153261

**Published:** 2025-08-04

**Authors:** Marek Zdaniewicz, Robert Duliński, Jana Lakatošová, Janusz Gołaszewski, Krystyna Żuk-Gołaszewska

**Affiliations:** 1 Department of Fermentation Technology and Microbiology, Faculty of Food Technology, University of Agriculture in Krakow, Balicka Street 122, 30-149 Krakow, Poland; marek.zdaniewicz@urk.edu.pl; 2 Department of Biotechnology and General Food Technology, Faculty of Food Technology, University of Agriculture in Krakow, Balicka Street 122, 30-149 Krakow, Poland; 3 AgroBioTech Research Centre, Slovak University of Agriculture in Nitra, Tr. A. Hlinku 2, 949 76 Nitra, Slovakia; jana.lakatosova@uniag.sk; 4 Faculty of Health Sciences, University of Lomza, 14 Akademicka St, 18-400 Łomża, Poland; jgolaszewski@al.edu.pl; 5 Department of Agrotechnology and Agribusiness, Faculty of Agriculture and Forestry, University of Warmia and Mazury in Olsztyn, Oczapowskiego Street 8, 10-719 Olsztyn, Poland; kzg@uwm.edu.pl

**Keywords:** *Cannabis sativa* L., malting, polyphenols, vitamins, cannabinoids, functional foods

## Abstract

The incorporation of Cannabis sativa L. seeds into barley wort was investigated to enhance the functional profile of beer. Hemp seeds (cv. Henola) were malted via controlled steeping, germination, and kilning, then added to barley malt at 10% and 30% (*w*/*w*) in both malted and unmalted forms. Standard congress mashing produced worts whose physicochemical parameters (pH, extract, colour, turbidity, filtration and saccharification times) were assessed, alongside profiles of fermentable sugars, polyphenols, B-group vitamins, and cannabinoids. Addition of hemp seeds reduced extract yield without impairing saccharification or filtration and slightly elevated mash pH and turbidity. Maltose and glucose levels declined significantly at higher hemp dosages, whereas sucrose remained stable. Wort enriched with 30% unmalted seeds exhibited the highest levels of trans-ferulic (20.61 µg/g), gallic (5.66 µg/g), trans-*p*-coumaric (3.68 µg/g), quercetin (6.07 µg/g), and trans-cinnamic (4.07 µg/g) acids. Malted hemp addition enhanced thiamine (up to 0.302 mg/mL) and riboflavin (up to 178.8 µg/mL) concentrations. Cannabinoids (THCA-A, THCV, CBDV, CBG, CBN) were successfully extracted at µg/mL levels, with the total cannabinoid content peaking at 14.59 µg/mL in the 30% malted treatment. These findings demonstrate that hemp seeds, particularly in malted form, can enrich barley wort with bioactive polyphenols, vitamins, and non-psychoactive cannabinoids under standard mashing conditions, without compromising key brewing performance metrics. Further work on fermentation, sensory evaluation, stability, and bioavailability is warranted to realise hemp-enriched functional beers.

## 1. Introduction

The use of plant-based raw materials in the search for new products with high nutritional value and appealing sensory properties aligns with current trends in food innovation in the 21st century [1]. Among the many “rediscovered” crop plants, *Cannabis sativa* subsp. *sativa* holds a special place [2]. Its seeds are characterised by a unique chemical composition, including a complete set of exogenous amino acids, a high content of unsaturated fatty acids, and a rich profile of polyphenols and cannabinoids [3,4]. These compounds exhibit anti-inflammatory, immunomodulatory, and neuroprotective properties, as confirmed by both in vitro and in vivo studies [5].

Hemp seeds are a functional food ingredient used not only in the production of flour and bakery products but also in the manufacturing of beverages such as beer [5,6].

Despite the growing recognition of hemp as a raw material for the food and pharmaceutical industries—with a compound annual growth rate (CAGR) of 17.1% for the hemp market from 2023 to 2030—its application in fermentation biotechnology remains underexplored [7].

The potential to enhance the functionality of beer products through the addition of *Cannabis sativa* seeds is based on their rich nutritional profile—including proteins, fermentable sugars, polyphenols, and cannabinoids—which collectively contribute to a unique taste and aroma [4]. This is further supported by the botanical relationship between hemp and hops, both of which belong to the *Cannabaceae* family [8].

As research has shown, beers with the addition of hemp seeds are characterised by a shorter filtration time, which may be due to the specific properties of fibres in hemp malt [3]. Moreover, the fermentation temperature has a significant impact on the sensory profile—fermentation at low temperatures results in beer with a fresh, clean taste, while fermentation at higher temperatures contributes to the development of fruity and floral aromas [8].

The first commercial beers with added hemp were already produced in 2017 [7]. In Europe, they are known under such trade names as Cannabeer, Klosterbrauerei Weissenohe, Green Times Brewing, and Lancaster. These beers were produced using hemp seeds or oil, and their alcohol content ranged from 4.1% to 6.0%, with or without Δ^9^-THC and CBD [7].

Hemp is rich in cellulose, and its extracts contain mainly glucose and xylose—a hemicellulose sugar that is not utilised by conventional brewing yeast [9]. Consequently, selecting appropriate processing methods and optimising the production cost of hemp beer represent current technological challenges.

The *Cannabis sativa* plant contains over 500 identified phytochemical compounds, including at least 120 cannabinoids. The most well-known include Δ^9^-tetrahydrocannabinol (Δ^9^-THC), Δ^8^-tetrahydrocannabinol (Δ^8^-THC), CBD, CBC, CBG, and CBND [5,9]. As far as beer aroma is concerned, one of the key volatile compounds identified in hemp seeds is α-terpinolene.

In our previous project, we confirmed the potential of malted hemp seeds as a raw material in the fermentation industry [10]. While most of the health benefits of industrial hemp have been studied in preclinical settings, it is essential that the industry pays close attention to dosing during manufacturing processes.

This study aimed to evaluate the impact of malted and unmalted hemp seeds on the bioactive profile and brewing-relevant properties of barley wort to support the development of functional beer.

The obtained results will also allow for the evaluation of the impact of hemp malt application on the functional and sensory properties of wort. Appropriate dosing during processing will help optimise health-promoting effects while mitigating potential safety risks associated with high concentrations of bioactive phytochemicals. It will also support the sustainable use of plant resources [11].

## 2. Materials and Methods

### 2.1. Materials

Hemp seeds of the Henola variety were used. The seeds were cultivated in Poland and delivered for research by Hemp Farm Poland (Krakow, Poland). Barley grains, malted by Ireks (Kulmbach, Germany), were used as a reference.

### 2.2. Brief Characterisation of cv. Henola and Phytochemical Profile of Hemp Seeds

Henola is a Polish variety of industrial hemp (*Cannabis sativa* var. *sativa*), cultivated primarily for seed production. This variety exhibits a seed yield of 1.5–2.1 t ha^−1^ and a straw yield of up to 10 t ha^−1^. The 1000-seed weight ranges from approximately 14 g, with protein content of 20–22% and fat content of 30–31% dry matter. The variety contains trace amounts of Δ^9^-THC in the dry weight of inflorescences (<0.2%) and CBD content of approximately 1%. Henola is characterised by early to very early flowering and seed maturation, with a vegetation period of 115–120 days after sowing (DAS) and a flowering period of 48–55 DAS. The plants are relatively short (170–200 cm) and resistant to lodging and exhibit satisfactory plant health.

Raw hemp seeds exhibit a complex phytochemical profile that includes various bioactive compounds, with cannabinoids being of particular interest, despite their generally low concentrations in the seed material itself. The cannabinoid content in hemp seeds is primarily the result of external contamination from resinous trichomes during harvesting and processing rather than natural biosynthesis within the seeds [12] (Farinon et al., 2020). Industrial hemp seeds typically contain very low levels of cannabinoids, with Δ^9^-tetrahydrocannabinol (THC) concentrations ranging from 0.06 to 5.91 μg/g and cannabidiol (CBD) from 0.32 to 25.55 μg/g [13]. However, these concentrations can vary significantly, depending on the hemp variety, extraction method, and processing conditions. Beyond cannabinoids, hemp seeds are rich in phenolic compounds, particularly lignanamides and hydroxycinnamic acid amides [14,15]. These compounds, along with ferulic acid, *p*-coumaric acid, and syringic acid, contribute significantly to the antioxidant properties of hemp seeds [16].

### 2.3. Malting Procedure

The process of malting hemp seeds was carried out under laboratory conditions. The process consisted of three main stages: soaking the grain, germination, and drying. The total soaking time lasted six hours and consisted of nine water and nine air cycles. For this purpose, 100 g of seeds was soaked in 500 mL of water at 19 °C for 30 min, then the water was removed, and the seeds were left out for five minutes while being constantly shaken. After completing the required number of soaking cycles, the samples were weighed and transferred for germination. During germination, the soaked seeds were placed at a temperature of 20 °C. Every 24 h, their surface was sprinkled, and the entire batch was mixed. The total germination period lasted 96 h, after which the germinated seeds were dried at 50 °C for 5 h to the desired moisture level (below 4% m/m).

#### 2.3.1. Preparation of Laboratory Wort

##### Mashing

Brewing worts with varying additions of hemp seeds (0%—control; 10% hemp seed malt—trial 1; 30% hemp seed malt—trial 2; and 30% unmalted hemp seeds—trial 3) were prepared using a method of handling barley malt (Analytica European Brewery Convention (EBC 4.5.1)). For this purpose, 50 g of grains milled in a laboratory grinder was weighed into tarred mash containers, which were then placed in a water-heated apparatus at 45 °C. Agitators were set up, and the “Congress program” was selected. Next, 200 mL of distilled water at 45 °C was poured in portions into the containers. The apparatus was held at 45 °C for 30 min. Then, the temperature was raised at the rate of 1° C/min until it reached 70 °C, with constant stirring of samples. When the apparatus reached 70 °C, 100 mL of distilled water warmed to the same temperature was added to the cups, and then the set temperature was maintained for 1 h. Next, the containers were cooled to 20 °C and filled up with distilled water to the mass of 450.0 g and filtered through a paper filter. In order to ensure high clarity, the first portions of the filtrate were recirculated. Subsequently, after filtration, the main wort’s parameters (extract, colour, fermentability) were measured in accordance with European Brewery Convention (EBC) methods 8.3, 8.5, and 8.6.1.

#### 2.3.2. RP-HPLC-DAD Analysis of the Selected Polyphenol Compounds

##### Polyphenols Determination

Single-component standard stock solutions were prepared by dissolving approximately 5 mg of each compound (Sigma-Aldrich, Chemie GmbH, Steinheim, Germany) in 10 mL of methanol (HPLC grade), followed by filtration through PVDF syringe filters (0.45 µm, 13 mm diameter; Frisenette ApS, Knebel, Denmark) into the HPLC vials. The calibration standard solutions were then prepared by diluting the stock solutions with methanol, within the concentration ranges shown in Table 1.

Each wort sample (~45 mL) was lyophilised and analysed by HPLC-DAD to evaluate the content of selected polyphenols, according to [17,18], with modification. An Agilent Infinity 1260 system equipped with a diode array detector (Agilent Technologies Inc., Waldbronn, Germany) was used. All analyses were performed on an Accucore C18 column (100 × 4.6 mm, 2.6 µm; Thermo Fisher Scientific Inc., Waltham, MA, USA). The sample was prepared at a concentration of 4 mg/mL in a mixture of mobile phases (A:B, 93:7). Mobile phase A consisted of approximately 0.1% phosphoric acid (ACS reagent, ≥85.0%; Merck KGaA, Darmstadt, Germany), while mobile phase B used acetonitrile (HPLC grade; Merck KGaA, Darmstadt, Germany) acidified with 1 mL of phosphoric acid per litre of acetonitrile. Double deionised water (ddH_2_O) was treated (18.2 MΩ cm^−1^) using a Simplicity 185 purification system (Millipore S.A.S, Molsheim, France). A gradient elution programme was used, again according to [17], with a flow rate of 0.6 mL/min. Each sample was prepared and analysed in duplicate (*n* = 4).

##### Cannabinoids Determination

Cannabidivarin (CBDV), tetrahydrocannabivarin (THCV), cannabidiol (CBD), delta9-tetrahydrocannabinolic acid (THCAA), cannabigerol (CBG), cannabinol (CBN), and cannabichromene (CBC) were supplied by Sigma-Aldrich. Single-component standard stock solutions were prepared by dissolving 1 mL of each compound (1 mg/mL) in 10 mL of methanol (HPLC grade). A working solution at a concentration of 10 µg/mL was immediately prepared by diluting the stock standards with methanol, then stored at −20 °C until analysis. Calibration curves were prepared at concentrations of 10, 20, and 30 ng/mL.

An aliquot of 1 mL wort was extracted with 2 mL of hexane/isopropanol (9:1), following the method of Pellegrini et al., 2005 [19], with modification. The mixture was homogenised on a shaker for 1 h and centrifuged at 4000 rpm for 5 min. The organic layer was separated into a new tube, and the sediment was re-extracted. The combined organic layers were evaporated to dryness under a nitrogen stream. The dried samples were dissolved in methanol, and a 1 µL amount was injected for analysis.

Analyses were performed in triplicates using gas chromatography with a flame-ionisation detector (GC-FID) (7890A; Agilent Technologies Inc., Waldbronn, Germany), according to the method of Janatová et al., 2022 [20,21], with modification. The helium flow rate was set to 1.2 mL/min, the injector temperature was maintained at 280 °C, and the injection volume was 1.0 µL.

Qualitative analyses were performed using gas chromatography coupled with a mass spectrometry detector (GC-MSD) (7890B-5977A; Agilent Technologies Inc., Waldbronn, Germany) and a CombiPal autosampler 120 (CTC Analytics AG, Zwingen, Switzerland), under the same conditions. The mass detector parameters were as follows: ionisation energy of filament: 70 eV, transfer line temperature: 250 °C, MS source temperature: 230 °C, quadrupole temperature: 150 °C. The mass spectrometer was programmed under electron impact (EI) in a full-scan mode at *m*/*z* 40–400. The identification of compounds was carried out by comparing mass spectra (over 80% match) with a commercial database NIST^®^ 2017. Each sample was measured in triplicate.

#### 2.3.3. Analysis of the Fermentable Sugars

Fermentable sugars were determined according to the method recommended in Analytica-EBC 2.27, with minor modifications (1), using a Dionex Ultimate-3000 HPLC apparatus equipped with an autosampler with eluent preconditioning, an Aminex HPX-87C column (300 × 7.8 mm^2^), and a refractometer (Knauer, Berlin, Germany, model 2400i). The chromatographic conditions were as follows: injection volume, 5 µL; mobile phase, deionised water (18 MΩ); flow rate, 0.5 mL/min; thermostatisation, 85 °C; and detection in positive polarity mode [2].

#### 2.3.4. Analysis of the B Group Vitamins

Detection of vitamins B1 and B2 was performed as described by Starzyńska-Janiszewska et al. [22], with modification for thiamine detection. The separation of riboflavin and vitamin B1 (as thiochrome) was performed using reversed-phase high-performance liquid chromatography (Luna C18, 250 mm × 4 mm i.d.; Phenomenex, Torrance, CA, USA) isocratically, with a mobile phase consisting of methanol and 0.05 M sodium acetate (30:70 *v*/*v*) at a flow rate of 1 mL/min. The fluorimetric detector was set to excitation wavelengths of 366 and 422 nm and emission wavelengths of 435 and 522 nm for vitamins B1 and B2, respectively. The conversion of thiamine to thiochrome was performed post-column, following modification with an oxidising reagent (0.1% potassium hexacyanoferrate (III) in 12% sodium hydroxide), delivered by a peristaltic pump at a flow rate of 0.2 mL/min.

### 2.4. Statistical Analysis

Experimental data were subjected to a one-way analysis of variance (ANOVA) to detect significant differences among means, and results are expressed as mean ± standard deviation (SD). Differences among means were checked by the Tukey or HSD test at *p* < 0.05 using Statistica for Windows, version 13.0 (StatSoft Inc., Tulsa, OK, USA) statistical software.

## 3. Results and Discussion

### 3.1. Analysis of the Physicochemical Parameters of the Wort

Table 2 presents the basic physicochemical properties of wort obtained from barley malt (control) and from wort with the addition of 10% and 30% hemp seeds malt, as well as 30% unmalted hemp seeds. The analysis includes parameters crucial for assessing the quality of the wort and its suitability for subsequent stages of beer production, including pH, extract content, colour, turbidity, filtration time, and wort’s fermentability.

The research methodology was based on standard congress wort in accordance with the guidelines of the European Brewery Convention (EBC). In addition to barley malt, other raw materials are often used in beer wort production. These materials are commonly referred to as adjuncts [23]. Their inclusion in a beer recipe depends on the function they serve. It may have an impact on increasing production profitability and/or enhancing the product with higher levels of bioactive compounds [24]. The use of hemp seeds malt and raw hemp seeds did not have a negative effect on the wort production process (Table 2). Neither saccharification nor filtration times were prolonged, which is often the case with other adjuncts (e.g., buckwheat) [23]. The saccharification times observed were typical of standard congress wort, while the filtration time was improved in the case of a higher proportion of the new raw material. However, it is worth noting that the use of both hemp seeds malt and raw hemp seeds did not enhance extract recovery from the raw material, nor did it improve the wort’s fermentability, as is observed with certain malt substitutes. The addition of 10% hemp seeds malt reduced the extract content by 5%. In turn, a 30% proportion of malted and unmalted hemp seeds resulted in reductions in extract values of 23% and 24%, respectively. Under control conditions, the extract level was characteristic of barley malt wort and comparable to that of tritordeum malt wort [25]. A reduction in wort extract with the addition of 30% hemp seeds was demonstrated. A similar trend was observed by Zdaniewicz et al., 2021 [26], who found that increasing the proportion of oats in the grist to 50% resulted in a comparable reduction in extract to that seen with 30% hemp seeds. As shown in previous studies [27], lower extract values were significant due to their direct impact on the amount of alcohol produced during fermentation, as well as on the development of the beer’s full flavour profile. Therefore, unlike other adjuncts such as corn or unmalted barley [27], the addition of hemp seeds did not improve production profitability. However, it may confer other beneficial properties to the final product. The wort without the addition of hemp seeds (control) was characterised by a higher pH of 5.9. Increasing the proportion of hemp seeds in the wort resulted in a further increase in pH (5.95). In cases when corn or rice was used in beer production, significantly lower pH values were reported—5.4 and 5.6, respectively—compared to those observed in the study [28]. Nevertheless, obtaining higher pH values does not pose a significant production challenge, as in most cases, it is suggested to adjust the mash pH during the mashing process. As demonstrated by Ivanov et al., 2023 [29], the correct mash pH was achieved by the addition of lactic acid or acidified malt.

The raw materials used during the mashing stage have a significant impact on the colour of the final product—beer. All the wort colour values obtained were characteristic of light malts and were significantly lower compared to those produced using other types of malt (e.g., caramel malt) [29]. Among all the variants analysed, only the beer brewed with the addition of 10% hemp seeds malt exhibited the highest colour value, amounting to 5.73 EBC (Table 2). The intensity of the colour did not increase with the addition of 30% hemp seeds, and its value was significantly lower compared to the control. This difference may be explained by the significantly lower extraction of all components (including coloured ones) in samples with a higher proportion of the new raw material, which is also reflected in the lower extract levels obtained. A similar trend was observed in the wort turbidity analysis, where the wort with a 10% addition of hemp seeds exhibited the highest value of the parameter tested.

However, the values of the analysed sugars differed. Under control conditions, the contents of maltose and glucose were 51.23 and 14.02 mg/mL, respectively, aligning with the findings of Laureys et al., 2023 [23], but differing significantly from most other adjuncts tested. Meanwhile, the use of 10% malted hemp seeds resulted in a decrease in maltose content by 13%. In contrast, the addition of 30% raw or malted hemp seeds did not significantly affect the maltose content, suggesting that the malting process had no influence on the content of the main wort sugar (maltose). Under control conditions and with the 10% hemp seeds addition, the glucose contents were 14.03 g/L and 13.42 g/L, respectively. These values differed significantly from the samples containing 30% of the raw material, which exhibited comparable levels to each other (10.17–10.55 mg/mL) (Table 3). This decrease may be partially attributed to the inherently low content of simple sugars in hemp seeds, as reported by Galasso et al., 2016 [30], who demonstrated that carbohydrates in hemp seeds are primarily stored in the form of fibre and non-fermentable polysaccharides, with relatively minor amounts of glucose, fructose, or sucrose. In contrast, the highest proportion of hemp seeds in the wort did not affect the sucrose content, which remained between 2.74 and 3.04 mg/mL (Table 3). Analysis of the process and quality parameters during the wort production stage revealed that the addition of hemp seeds malt significantly reduced process efficiency. Therefore, detailed monitoring of the remaining components—particularly the bioactive ones—is crucial to demonstrate the potential of the tested seeds for the production of beverages based on beer wort.

### 3.2. Polyphenols and Selected B Vitamins Content

#### 3.2.1. Profile of Phenolic Compounds in Wort with Hemp Seeds

The profile of phenolic compounds included the contents of gallic acid, 4-hydroxybenzoic acid, catechin, *trans*-*p*-coumaric acid (trans isomer), *trans*-ferulic acid, quercetin, and *trans*-cinnamic acid in wort with the addition of hemp seeds. Our study showed that *trans*-ferulic acid had the highest content among all the phenolic compounds across all analysed wort variants, and its level increased with the addition of hemp seeds. In the control variant, its concentration amounted to 7.22 µg/g. The addition of 10% malted hemp seeds increased this value to 11.88 µg/g, while the use of 30% malted hemp seeds led to a further increase, reaching between 15.68 and 20.20 µg/g (Table 4). The content of this polyphenolic compound is consistent with previous research results on plant-based additives used to enrich fermented beverages with antioxidant compounds [13,31]. At the same time, the high content of ferulic acid, known for its antioxidant and anti-inflammatory properties, confirms the functional potential of hemp seeds as an additive to raw materials for brewing, enhancing the health-promoting qualities of beer [31].

Gallic acid was detected in wort with a 30% addition of malted hemp seeds and in wort with a 30% addition of unmalted hemp seeds, with content ranging from 3.06 to 5.10 µg/g and 4.64 to 5.66 µg/g, respectively. In contrast, 4-hydroxybenzoic acid was only detected in wort containing 30% unmalted hemp seeds (Table 3). Our research findings are consistent with the previous research by Zdaniewicz et al., 2024 [10] and Farinon et al., 2020 [12] on the possible degradation of this compound during the malting process. Similar results have been reported in studies investigating the interaction of phenolic compounds with the raw material matrix during fermentation [32].

A similar pattern was observed for the content of *p*-coumaric acid (2.72–4.19 µg/g) and cinnamic acid (4.07 ± 0.09 µg/g) in wort with 30% unmalted hemp seeds (Table 3). The presence of quercetin and selected phenolic acids in wort with the addition of unmalted hemp seeds is characteristic, which may suggest their higher natural content in plant material that has not been subjected to enzymatic processing. The degradation of these compounds during thermal and enzymatic treatment has been confirmed in studies by Shahidi et al. [31].

Gallic acid was detected at relatively low concentrations in the control samples; however, it was not quantified in the samples containing a 10% addition of malted hemp seeds. Notably higher levels of gallic acid were observed only in the samples with a 30% addition of malted (3.06–5.10 µg/g) and unmalted hemp seeds (4.64–5.66 µg/g). These findings indicate that the occurrence of gallic acid in wort is strongly influenced by the higher proportion of hemp-derived raw material.

Catechin hydrate was identified only in one control sample (42.64 ± 22.9 µg/g) and was not detected in any of the samples containing hemp addition. The presence of catechin may be attributed to heterogeneity in the malt material. Its absence in the hemp-supplemented samples could suggest dilution, degradation, or an influence of hemp components on the extraction efficiency of this compound.

It can be assumed that the malting process negatively affects the stability or extractability of certain phenolic compounds, resulting in their lower content in the finished wort. This was confirmed by Farinon et al., 2022 [33], who documented an approximately 20% decrease in antioxidant potential during the malting process.

On the other hand, chlorogenic acid, *trans*-caffeic acid, epicatechin, rutin, resveratrol, and kaempferol were not detected in any of the analysed wort variants.

#### 3.2.2. Content of Selected B Vitamins

In the context of the growing interest in fermented beverages enriched with plant raw materials with high bioactive potential, it is important to monitor the stability and content of vitamins that naturally occur in the components used to produce wort, such as barley malt or hemp seeds [5,34].

Thiamine (vitamin B1) and riboflavin (vitamin B2) play important roles in cellular metabolism. They act as enzymatic cofactors in carbohydrate metabolic pathways, taking part in the synthesis of nucleotides and steroid hormones and in nerve conduction [18,35]. The presence of these vitamins in raw materials may have both technological and nutritional significance. The first aspect results from the presence of vitamin B2 as both a colouring agent in beer and a photolabile compound, the derivative of which, under exposure to light, may imply transformations of one of the hop isohumulones, contributing to undesirable changes in the sensory properties of the final product [36].

Simultaneously, vitamin B1, as a key cofactor in metabolic pathways involving dehydrogenase and pyruvate decarboxylase, influences the efficiency with which yeast utilises substrates present in raw materials and semi-finished products [18]. Another aspect is the limited durability of these vitamins, particularly thiamine, but also riboflavin to a lesser extent—as they are sensitive to environmental factors such as high temperature, UV radiation, and the presence of oxidants [36,37]. Therefore, during technological processes—especially mashing and wort boiling—their partial degradation may occur. This was previously demonstrated in studies on the stability of vitamins in cereal products [18,38].

The addition of hemp seeds increased the riboflavin (RFL, vitamin B2) content in the wort, with the effect being more pronounced in the case of malted seeds. The highest RFL concentration (178 µg/mL) was recorded in the wort containing 30% malted hemp seeds. This value was nearly 26% higher than that of the control variant and significantly higher than all other variants analysed (*p* = 0.000199). On the other hand, the addition of 30% unmalted hemp seeds increased the RFL content by only 4.5% (Table 5). Our findings are supported by the research of Sirangelo et al., 2025 [38] which demonstrated that the malting of hemp seeds enhances the release of RFL during mashing due to the interaction between malt enzymes and bioactive compounds present in the seeds.

The wort prepared solely from barley malt (control) was characterised by the lowest average thiamine (B1) level (0.28123 mg/mL). The malting of hemp seeds significantly influenced B1 availability in the finished wort, with the addition of 10% malted hemp seeds leading to a significant increase in B1 content to 0.29341 mg/mL—an increase of approximately 4.3% compared to the control. The addition of 30% malted hemp seeds resulted in a further significant increase in vitamin B1 content by almost 7.4% (0.30199 mg/mL). In turn, the wort containing 30% unmalted hemp seeds had an average B1 content of 0.28867 mg/mL. However, this difference was not statistically significant (*p* = 0.111100).

The obtained results showed that the addition of malted hemp seeds, particularly at 30% of the grist, increased the B1 content in the wort. This may be attributed to the activity of the enzyme cocktail during the malting process, which promotes the release of B1 from phosphate derivatives and protein complexes within the cell matrix [22,35]. An additional benefit of the presence of this vitamin in the wort is its release during malting, which not only enhances the nutritional value of the beer produced but also stimulates yeast metabolism at the fermentation stage [39].

However, the obtained data show that the effect of adding hemp seeds, both malted and unmalted, although statistically significant, especially at a 30% share, is disproportionate to the difference in the content of these vitamins in the initial raw material (barley: 0.43 mg/100 g; hemp seeds: 1.4 mg/100 g). This may indicate incomplete extraction of vitamins from the hemp seeds—particularly vitamin B1—under the standard wort mashing conditions [5,18].

The results obtained in our research are consistent with previous studies on the enrichment of fermented products using plant raw materials with health-promoting properties [22,40]. Further studies are necessary to assess the stability of vitamins during the fermentation and storage of beer, as well as their bioavailability in in vitro and in vivo tests [41].

### 3.3. Cannabinoid Profile in Hemp Seeds-Enriched Wort

The inclusion of hemp seeds in the barley wort recipe results in the incorporation of bioactive compounds naturally present in the plant, including cannabinoids, flavonoids, sterols, and tocopherols. The mashing process, a key stage in beer brewing, facilitates the transfer of these compounds from the raw material to the aqueous matrix, as confirmed by recent studies by Padilla-González et al., 2023 [15] and Tănase Apetroaei et al., 2024 [4]. Assessing the efficiency of the transfer of bioactive components at the mashing stage is important from the perspective of both functional beverage development and technological and regulatory considerations. A deeper understanding of the process allows for the optimisation of technological parameters to maximise the extraction of desired compounds and ensure compliance with legal standards regarding cannabinoid content [42]. The final product—beer or a barley wort-based beverage enriched with hemp seeds—is characterised by a unique sensory profile and the presence of biologically active compounds. It combines the traditional qualities of barley beer with potential health benefits associated with hemp-derived ingredients.

Table 6 contains the mean and standard deviation of cannabinoids concentrations. The analysis of results reveals a selective cannabinoid extraction pattern, with five out of seven tested compounds successfully detected in hemp-enriched wort samples. THCA-A was the predominant cannabinoid across all treatments, with concentrations ranging from 9.477 µg/mL (30% unmalted hemp seeds) to 10.537 µg/mL (30% malted hemp seeds). The second most abundant compound was THCV, with concentrations between 2.267 µg/mL (10% malted hemp seeds) and 2.790 µg/mL (30% unmalted hemp seeds). At moderate levels, CBDV ranged from 0.557 to 0.630 µg/mL across treatments, while the lowest concentrations detected were CBG (0.350–0.430 µg/mL) and CBN (0.217–0.333 µg/mL). CBC and CBD were not detected in any samples.

The 10% malted hemp seeds treatment yielded a total cannabinoid concentration of 13.601 µg/mL, with THCA-A comprising approximately 74.5% of the total cannabinoid content. The relatively high standard deviations for THCA-A (±3.048) and THCV (±1.270) suggest some variability in extraction efficiency at this concentration level.

The 30% malted hemp seeds treatment produced the highest total cannabinoid concentration at 14.591 µg/mL, representing a 7.3% increase over the 10% treatment. This improved extraction efficiency demonstrates a dose-dependent relationship, with more consistent results indicated by lower standard deviations across most compounds.

The 30% unmalted hemp seeds treatment yielded 13.610 µg/mL total cannabinoids, slightly lower than the 30% malted treatment but comparable to the 10% malted treatment. The lower standard deviations suggest more consistent extraction from unmalted seeds, particularly for THCA-A (±0.802).

Comparing the 30% malted and unmalted treatments reveals differential extraction patterns: malting appears to enhance the recovery of certain cannabinoids (e.g., THCA-A) while potentially diminishing others (e.g., THCV), likely due to enzymatic transformations during the malting process. These findings corroborate research by Farinon et al., 2022 [33], who demonstrated that malting can reduce certain bioactive compounds by approximately 20% while enhancing others.

The successful extraction of cannabinoids during mashing is noteworthy given thermal stability considerations. THCA-A begins significant decarboxylation at approximately 104 °C, well above the mashing temperature of 70 °C, preserving the acidic forms. Research by Maly et al., 2024 [43] reported an average transfer rate of only 0.5% for Δ^9^-THC into tea infusions, underscoring the relatively efficient extraction observed in wort (µg/mL levels) compared to aqueous infusion preparations.

Overall, these findings provide quantitative evidence that cannabinoids are effectively extracted from hemp seeds into barley wort under standard mashing conditions. The detected concentrations, though modest, represent meaningful levels of bioactive compounds suitable for functional beverage applications. The absence of CBD and CBC, alongside the prevalence of minor cannabinoids such as THCV and CBDV, aligns with the characteristic profile of industrial hemp seeds used in food ingredients, as documented by multiple researchers, including Tănase Apetroaei et al., 2024 [4] and Farinon et al., 2022 [33].

**Figure 1:**
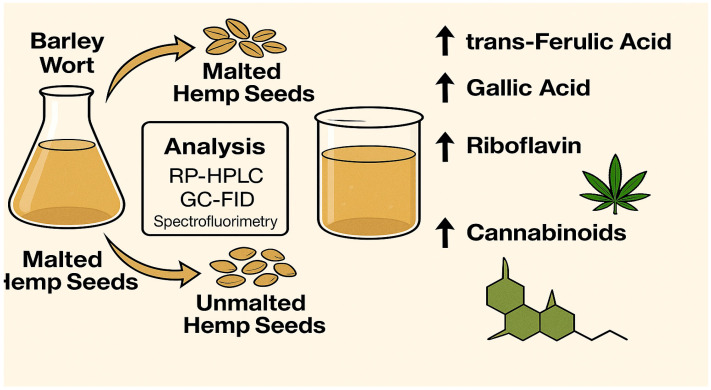
Graphical summary of bioactive enrichment trends in barley wort supplemented with malted and unmalted *Cannabis sativa* L. seeds.

In summary, the cannabinoid levels detected in hemp-enriched barley wort (0.217–10.537 µg/mL) correspond to the lower end of doses found in commercial cannabis-infused beverages, balancing regulatory safety margins and functional, non-psychoactive dosing [30,42]. Thus, hemp seed incorporation can enrich barley wort with cannabinoids while maintaining compliance with industrial hemp standards, supporting the development of functional brewing applications (Figure 1).

## 4. Conclusions

This study comprehensively evaluated the effect of incorporating malted and unmalted *Cannabis sativa* seeds into barley wort on its physicochemical properties and bioactive compound profile. The results demonstrated the following:

The addition of hemp seeds, particularly in unmalted form, significantly enriched the wort in polyphenolic compounds—most notably, *trans*-ferulic and gallic acids—known for their antioxidant and anti-inflammatory activity.

The presence of malted hemp seeds improved the release of B-group vitamins, including thiamine and especially riboflavin, with the 30% malted hemp seeds variant showing the highest concentrations. This confirms the positive impact of malting on vitamin bioavailability.

The mashing process selectively transfers non-psychoactive cannabinoids from hemp seeds into barley wort, with Δ^9^-tetrahydrocannabinolic acid (THCA-A) comprising the majority (9.48–10.54 µg/mL), followed by Δ^9^-tetrahydrocannabivarin (THCV, 2.27–2.79 µg/mL), cannabidivarin (CBDV, 0.56–0.63 µg/mL), cannabigerol (CBG, 0.35–0.43 µg/mL), and cannabinol (CBN, 0.22–0.33 µg/mL); the total cannabinoid content peaks at 14.59 µg/mL in the 30% malted treatment, demonstrating dose-dependent extraction efficiency under standard mashing conditions while maintaining compliance with industrial hemp regulations. While increasing hemp content reduced extract yield, key brewing parameters—including wort pH, saccharification time, and filtration performance—remained stable or even improved.

Still, aspects like extended fermentation, storage stability assessments, or consumer sensory evaluations within a complete beer matrix are critical for fully validating the functional potential and market feasibility of hemp-enriched beers. To address these gaps, future research should investigate the fermentation behaviour of such worts, the bioavailability of key phytochemicals in finished beers, and long-term stability under realistic storage conditions. It is worth highlighting that a separate study focusing on the sensory and volatile compound profile of these hemp-supplemented worts is currently being prepared, which will provide complementary insights into their potential for commercial brewing applications.

## Figures and Tables

**Table 1:** Calibration curves (ng), wavelengths, and purity of selected polyphenol standards.

Compounds	CAL 1	CAL 2	CAL 3	R2 (%)	Wavelength (nm)	Purity of Standard
Catechin hydrate	13.269	26.538	53.077	99.972	240	≥96% (HPLC)
Epicatechin	3.846	7.692	15.385	99.986	240	≥98% (HPLC), from green tea
Chlorogenic acid	14.423	28.846	57.692	99.987	324	primary reference standard, 96.67%
Rutin	16.731	33.462	66.923	99.980	256	≥97.84%, reference standard
Quercetin	16.077	32.154	64.308	99.986	256	≥98%, HPLC
Resveratrol	15.231	30.462	60.923	99.984	320	≥99.2%, certified reference material TraceCERT^®^
Kaempferol	15.769	31.538	63.077	99.987	265	≥93.9%, primary reference standard
4-Hydroxybenzoic acid	19.231	38.462	76.923	99.981	256	≥99%
Trans-cinnamic acid	17.192	34.385	68.769	99.984	276	≥98%, analytical standard
Trans-*p*-coumaric acid	14.615	29.231	58.462	99.980	320	≥98.0%, (HPLC)
Trans-ferulic acid	17.00	34.00	68.00	99.988	324	≥99.4%, certified reference material TraceCERT^®^
Trans-caffeic acid	16.308	32.615	65.231	99.982	324	≥99.3%, certified reference material TraceCERT^®^
Gallic acid	17.154	34.308	68.615	99.981	276	certified reference material TraceCERT^®^

**Table 2:** Physicochemical properties of worts prepared with barley malt and varying proportions of malted and unmalted hemp seeds.

	Control	10% Hemp Seeds Malt	30% Hemp Seeds Malt	30% Unmalted Hemp Seeds
pH	5.86 ^a^ ± 0.05	5.95 ^b^ ± 0	6.01 ^c^ ± 0.01	5.96 ^b^ ± 0.01
Extract (% m/m)	8.57 ^a^ ± 0.12	8.03 ^b^ ± 0.05	6.63 ^c^ ± 0.06	6.5 ^d^ ± 0
Colour (EBC)	4.8 ^a^ ± 0.14	5.73 ^b^ ± 0.19	4.33 ^c^ ± 0.12	4.5 ^d^ ± 0
Turbidity (NTU)	64.4 ^a^ ± 2.77	92.8 ^b^ ± 5.98	19.04 ^c^ ± 1.49	20.37 ^c^ ± 0.9
Filtration time	60 ^a^ ± 14	60 ^a^ ± 15	40 ^b^ ± 6	50 ^b^ ±4
Saccharification time (min)	<10	<10	<10	<10
Fermentability (%)	64.96 ^a^ ± 1.1	62.39 ^b^ ± 0.8	58.82 ^c^ ± 0.4	60.23 ^bc^ ± 1.4

Note: Statistically significant differences are indicated by different letters in the same row (Tukey HSD test, *p* < 0.05).

**Table 3:** Average fermentable sugars content (mg/mL) in barley wort with the addition of malted and unmalted hemp seeds.

Sample	Maltose	Glucose	Sucrose
Control barley malt	51.234 ^a^	14.028 ^a^	2.742 ^b^
Barley malt with 10% of malted hemp seeds	44.334 ^b^	13.423 ^b^	3.045 ^a^
Barley malt with 30% of malted hemp seeds	35.390 ^c^	10.558 ^c^	2.396 ^c^
Barley malt with 30% of unmalted hemp seeds	34.874 ^c^	10.176 ^c^	2.435 ^c^

Note: Statistically significant differences are indicated by different letters (Tukey HSD test, *p* < 0.05).

**Table 4:** Selected polyphenols content (µg/g) in barley wort with the addition of malted and unmalted hemp seeds.

Sample	Gallic Acid	4-Hydroxybenzoic Acid	Catechin Hydrate	*Trans*-*p*-Coumaric Acid	*Trans*-Ferulic Acid	Quercetin	*Trans*-Cinnamic Acid
Control barley malt	0.81 ^a^ ± 0.06	ND	42.64 ± 0.52	ND	7.22 ^a^ ± 0.41	ND	ND
10% malted hemp seeds	ND	ND	ND	ND	11.81 ^b^ ± 0.39	ND	ND
30% malted hemp seeds	4.18 ^b^ ± 0.79	ND	ND	ND	17.62 ± 0.69 ^c^	ND	ND
30% unmalted hemp seeds	4.99 ^c^ ± 0.15	1.24 ± 0.02	ND	3.68 ± 0.10	20.61 ^d^ ± 0.55	6.07 ± 0.32	4.07 ± 0.24

ND—not detected. Chlorogenic acid, *trans*-caffeic acid, epicatechin, rutin, resveratrol, and kaempferol were also not detected in any of the tested wort samples. Statistically significant differences are indicated by different letters (Tukey HSD test, *p* < 0.05).

**Table 5:** Mean concentrations of thiamine (B1) and riboflavin (RFL) in barley wort with the addition of malted and unmalted hemp seeds.

Sample Name	Thiamine (B1) (mg/mL)	Riboflavin (RFL) (µg/mL)
Control barley malt	0.28123 ^a^	142.4 ^a^
10% malted hemp seeds	0.29341 ^b^	163.2 ^c^
30% malted hemp seeds	0.30199 ^b^	178.8 ^d^
30% unmalted hemp seeds	0.28867 ^ab^	148.4 ^b^

Note: Average concentrations of vitamins (µg/mL ± SD) in wort with the addition of hemp seed variants. Statistically significant differences are indicated by different letters (Tukey HSD test, *p* < 0.05).

**Table 6:** Cannabinoid concentrations (µg/mL ± SD) in barley wort with the addition of malted and unmalted hemp seeds.

Cannabinoid	Control Barley Malt	10% Malted Hemp Seeds	30% Malted Hemp Seeds	30% Unmalted Hemp Seeds
CBDV	ND	0.630 ± 0.078	0.557 ± 0.045	0.583 ± 0.059
THCV	ND	2.267 ± 1.270	2.737 ± 0.196	2.790 ± 0.182
CBC	ND	ND	ND	ND
CBD	ND	ND	ND	ND
THCA-A	ND	10.137 ± 3.048	10.537 ± 2.153	9.477 ± 0.802
CBG	ND	0.350 ± 0.238	0.427 ± 0.067	0.430 ± 0.062
CBN	ND	0.217 ± 0.188	0.333 ± 0.050	0.330 ± 0.044

## Data Availability

Data are contained within the article.
